# Integrating Convolutional Neural Networks with Attention Mechanisms for Magnetic Resonance Imaging-Based Classification of Brain Tumors

**DOI:** 10.3390/bioengineering11070701

**Published:** 2024-07-10

**Authors:** Zahid Rasheed, Yong-Kui Ma, Inam Ullah, Mahmoud Al-Khasawneh, Sulaiman Sulmi Almutairi, Mohammed Abohashrh

**Affiliations:** 1 School of Electronics and Information Engineering, Harbin Institute of Technology, Harbin 150001, China; zahid.rasheed@hit.edu.cn (Z.R.); yk_ma@hit.edu.cn (Y.-K.M.); 2 Department of Computer Engineering, Gachon University, Sujeong-gu, Seongman 13120, Republic of Korea; 3 School of Computing, Skyline University College, University City Sharjah, Sharjah 1797, United Arab Emirates; mahmoudalkhasawneh@outlook.com; 4 Applied Science Research Center, Applied Science Private University, Amman 11931, Jordan; 5 Jadara University Research Center, Jadara University, Irbid 21110, Jordan; 6 Department of Health Informatics, College of Public Health and Health Informatics, Qassim University, Qassim 51452, Saudi Arabia; ssmtiery@qu.edu.sa; 7 Department of Basic Medical Sciences, College of Applied Medical Sciences, King Khalid University, Abha 61421, Saudi Arabia; mabohashrh@gmail.com

**Keywords:** deep learning, brain tumors, magnetic resonance imaging (MRI), classification, healthcare, neural network, medical image

## Abstract

The application of magnetic resonance imaging (MRI) in the classification of brain tumors is constrained by the complex and time-consuming characteristics of traditional diagnostics procedures, mainly because of the need for a thorough assessment across several regions. Nevertheless, advancements in deep learning (DL) have facilitated the development of an automated system that improves the identification and assessment of medical images, effectively addressing these difficulties. Convolutional neural networks (CNNs) have emerged as steadfast tools for image classification and visual perception. This study introduces an innovative approach that combines CNNs with a hybrid attention mechanism to classify primary brain tumors, including glioma, meningioma, pituitary, and no-tumor cases. The proposed algorithm was rigorously tested with benchmark data from well-documented sources in the literature. It was evaluated alongside established pre-trained models such as Xception, ResNet50V2, Densenet201, ResNet101V2, and DenseNet169. The performance metrics of the proposed method were remarkable, demonstrating classification accuracy of 98.33%, precision and recall of 98.30%, and F1-score of 98.20%. The experimental finding highlights the superior performance of the new approach in identifying the most frequent types of brain tumors. Furthermore, the method shows excellent generalization capabilities, making it an invaluable tool for healthcare in diagnosing brain conditions accurately and efficiently.

## 1. Introduction

An abnormal cell that proliferates within brain tissues can result in the transformation of a brain tumor. Tumors are the second most common cause of mortality globally, as reported by the World Health Organization [1,2]. Brain tumors are primarily characterized as benign or malignant. Benign tumors are often not considered a significant threat to a person’s health. The main reasons include their inability to infiltrate neighboring tissues or cells, slower growth compared to malignant tumors, and limited spreading. In addition, recurrence after surgically removing benign tumors is typically rare.

On the other hand, malignant tumors tend to infect nearby organs and tissues more than benign tumors. They can significantly disrupt normal bodily functions if not treated swiftly and effectively. Early detection is crucial for the survival of patients with brain tumors, which are primarily classified into three forms: meningioma, glioma, and pituitary tumors. Moreover, meningioma and pituitary tumors are classified as benign, whereas glioma tumors are recognized as malignant. Furthermore, meningioma tumors arise from the meninges, the three layers of tissue covering the brain and spinal cord. Gliomas develop from ependymal cells, oligodendrocytes, and astrocytes, and pituitary tumors develop in the pituitary gland [3,4,5].

Consequently, it is crucial to discriminate between different tumor types to identify a patient precisely and select the most suitable treatment. Magnetic resonance imaging (MRI) is frequently used to identify various types of cancer despite the obstacles associated with human interpretation and managing huge quantities of data. Biopsies are commonly employed for the diagnosis and treatment of brain lesions.

However, the radiologist’s proficiency greatly impacts their ability to identify brain cancers quickly. Developing a diagnostic mechanism is essential for diagnosing cancers using MR imaging [6]. Implementing this method will maintain the objectivity of the diagnostic process and effectively decrease the chances of handed procedures. Artificial intelligence (AI) and machine learning (ML) have greatly revolutionized the healthcare industry [7,8,9,10,11]. The advent of technologies has brought forth innovative methodologies for radiologists in classifying MRI images, effectively tackling numerous health-related obstacles [12,13]. Medical imaging methods are acknowledged for their efficacy and are extensively used to identify cancer. The approach is significant because of its non-invasive nature, as it does not require intrusive processes [14,15].

Medical imaging is significant in healthcare, particularly for attaining all-inclusive visualization of brain tissue, which is essential in classifying brain tumors. The tumors vary in shape, size, and density. The tumors that appear similar may have different clinical characteristics. The large number of images in medical databases makes it complicated to classify MRI scans using neural networks effectively. Advances in generating MRI images from various perspectives could significantly increase the data sizes. In order to achieve better classification precision, the data must be preprocessed before feeding into different networks. CNNs are known for their robust characteristics, which include reduced preprocessing requirements and improved feature extraction abilities. Simpler network structures save resources during setup and training while increasing operating efficiency. Nonetheless, the use of these methods in clinical diagnostics and handheld tools may be limited by resource constraints. The appropriate approach is important for routine clinical evaluation of brain tumors.

The main contributions of this study are delineated as follows:

- This study presents a novel approach that combines hybrid attention with convolution neural networks to improve the efficiency of diagnosing glioma, meningioma, pituitary, and no-tumor cases.
- The objective of this study is to emphasize the effectiveness of the proposed method in comparison to previous studies, showcasing its capacity to provide effective results with fewer resources. Moreover, the method’s capacity for usage in a clinical research context is thoroughly evaluated.
- The findings from this study demonstrate that the proposed method surpasses the previous studies in terms of performance, as demonstrated on the benchmark dataset. Additionally, the study evaluates the prediction competencies of the framework by comparing it to pre-trained models, ultimately improving diagnostics methodologies and clinical necessities.

This article contains several sections. Section 2 of this study provides an overview of the literature. Section 3 highlights the dataset, methodology, and optimization approach. Section 4 presents the results derived from the experiments. Section 5 entails a discussion, and finally, Section 6 provides a conclusion.

## 2. Literature Review

Due to the above considerations, it may be difficult to distinguish between different forms of brain tumors. The authors explored the use of deep learning in the field of radiology, detailing the essential steps for implementing DL projects within this area. In addition, they explored the possible applications of DL in various medical sectors. Although DL has shown potential in some radiology applications, it is still not advanced enough to take over the roles played by radiologists [16,17]. However, there is potential for combining radiologists with deep learning procedures to improve diagnostic efficacy and precision. Various research approaches have been used to explore the effectiveness of MRI in the classification of brain tumors. Gumaei et al. proposed a strategy for classifying brain tumors that combines hybrid feature extraction techniques with RELM. The authors attained an accuracy of 94.23% by preprocessing brain images with min–max normalization and features extracted by the hybrid method and classifying them using the RELM method [18]. Srujan et al. constructed a deep learning system of sixteen CNN layers. This system included the Rectified Linear Unit (ReLU) as an activation function, and utilized the Adam optimizer within its architecture. The system attained a 95.36% accuracy rate, demonstrating its ability to classify various primary types of cancers [19]. Kaplan et al. introduced a novel classification method for identifying brain malignancies utilizing nLBP and αLBP for feature extraction. This approach particularly achieved a notable accuracy rate of 95.56% when combined with the nLBPD = 1 feature extraction method with the KNN classifier [14].

Huang et al. developed a CNNBCN network to categorize brain tumors. The method was evaluated using a randomly generated graph algorithm, which yielded an accuracy rate of 95.49% [20]. Deepak et al. employed a combination of CNN and SVM methods to categorize medical images depicting brain tumors based on a fivefold cross-validation method; the automated system demonstrated a notable accuracy rate of 95.82%, surpassing the performance of the state-of-the-art approaches [21]. Ghassemi et al. suggested a deep learning framework as a potential treatment method for classifying brain cancers. The framework extracted robust features from MRI images using pre-trained networks as GAN discriminators and achieved a 95.6% accuracy rate. In addition, the framework was involved in fivefold cross-validation, data augmentation, and dropout [22]. Ayadi et al. suggested brain tumor classification algorithms that included normalization, dense speeded-up robust features, and the histogram of gradient methods to improve the image quality and provide distinctive features. Additionally, the authors utilized Support Vector Machines (SVMs) as a classifier and attained a classification accuracy of 90.27% on the benchmarked dataset [23].

Noreen et al. reformed pre-trained networks, namely InceptionV3 and Xception, for classifying brain tumors. The models were combined with various ML classifiers, such as softmax, Random Forest, KNN, and SVM, and attained 94.34% accuracy with the InceptionV3 ensemble [24]. Ahmad et al. suggested a deep generative neural network as a classifier to categorize brain tumors. The method used generative adversarial networks combined with a variational auto-encoder to generate realistic tumor MRI images, which attained 96.25% accuracy [25]. Swati et al. proposed block-wise transfer learning to employ a pre-trained deep convolutional neural network (CNN) model. This approach was evaluated through 5-fold cross-validation using a representative dataset of T1-weighted images with minimal preprocessing approaches and eliminated manually designed features. The method attained an accuracy of 94.82% with VGG19, 94.65% with VGG16, and 89.95% with AlexNet [26]. Satyanarayana et al. proposed a method integrating CNN with mass correlation analysis (MCA). Initially, the Average Mass Elimination Algorithm (AMEA) removed unwanted noise. Subsequently, the CNN model was trained on these features, and MCA played a critical role in determining the weight measures assumed and maximizing the model performance. The strategy yielded an impressive 94% accuracy rate [27].

Deepak et al. proposed a class-weighted focal loss to solve the unbalanced training data problem in CNN-based tumor classification data. The authors investigated the effect of the loss on feature learning. They proposed two methods for improving the performance: majority voting, which involved aggregating classifier prediction from feature sets, and deep feature fusion, which involved combining features from CNNs trained using different loss functions. Furthermore, SVM and KNN models attained 94.9% and 95.6% accuracy, respectively, outperforming typical CNNs trained with cross-entropy loss [28]. Rezaei et al. introduced an integrated method for segmenting and classifying brain tumors using Figshare data. The methodologies encompassed feature extraction, noise reduction, Support Vector Machine (SVM)-based implementation for segmentation, and differentiation extraction (DE) selection. The classification of tumor slices was performed using WSVM, HIK-SVM, and KNN classifiers. When combined with MODE-based ensemble approaches, these classifiers demonstrated a precision rate of 92.46% [29].

## 3. Materials and Methods

**Figure 1:**
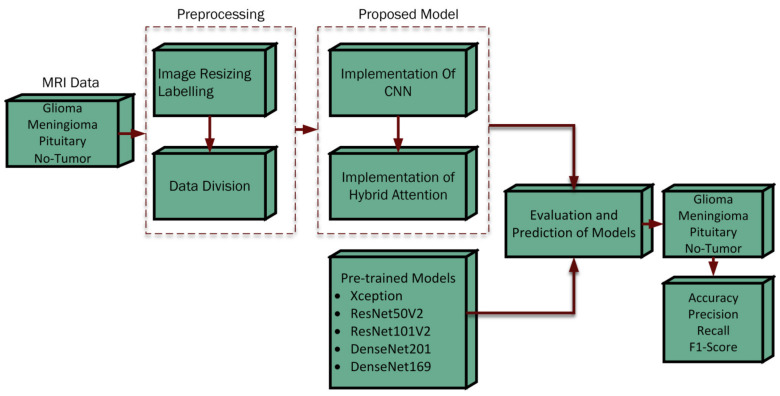
Procedural structure of the proposed framework.

The present study introduces an innovative methodology comprising several stages: The framework commenced by resizing the dimensions of the input data in order to achieve consistency in aspect ratio. Subsequently, a process of labeling was used to ensure a uniform distribution of data. The dataset was distributed into two subsets: 80% was used for training purposes, and the remaining 20% was reserved for testing. Following this, the model was trained through 5-fold cross-validation [30] using the Adam optimizer [31,32], which integrated callbacks for learning rate adjustment during the training procedure. Various metrics were employed to assess the efficacy of the model, including accuracy, precision, recall, and the F1-score, specifically for classification tasks. The procedural framework of the suggested methodology is illustrated in Figure 1.

### 3.1. Dataset

**Figure 2:**
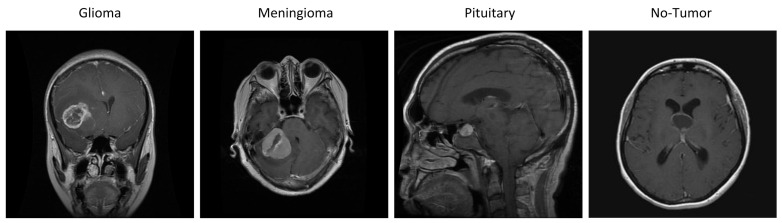
The different types of tumors contained in the dataset.

This study utilized an openly available MRI data set from the Kaggle repository [33]. The dataset integrates three publicly accessible sources: Figshare [34], SARTAJ [35], and BR35H [36]. It comprises 7023 grayscale and jpg format MRIs of the human brain, covering primary brain tumor types such as glioma, meningioma, and pituitary, as well as images without tumors. Figure 2 illustrates the various tumor types included in the dataset.

### 3.2. Proposed Architecture

**Figure 3:**
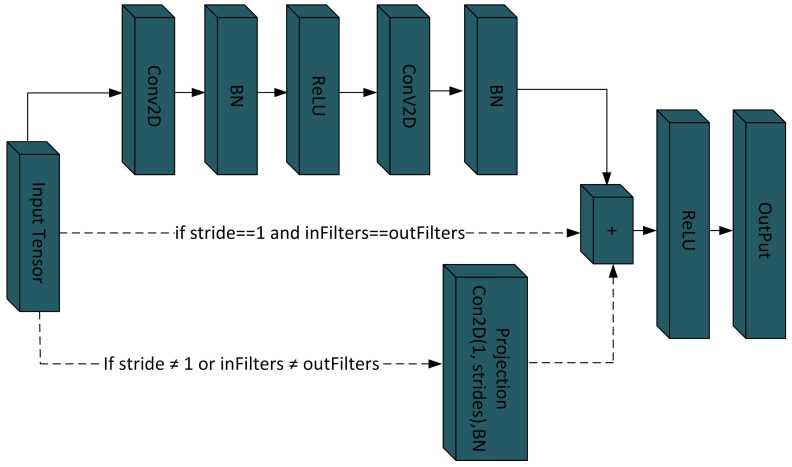
Illustration of the convolution blocks utilized in the suggested design.

In this study, a novel convolutional neural network is employed, incorporating advanced attention mechanisms to enhance the feature extraction for brain tumor classification. The proposed architecture consists of a convolutional block and a hybrid attention block. The convolutional block, as shown in Figure 3, is an integral module of the proposed model that comprises convolutional layers, batch normalization, ReLU, and skip connections. The block utilized two distinct convolutional operations, each followed by batch normalization (BN) [37], to optimize learning efficiency and model stability. Initially, input was processed through a convolutional operation using kernel size 3 × 3, stride size of 1, “same” padding, and L2 regularization (10^−3^), extracting spatial features while preserving the input dimension. The output was normalized using batch normalization (BN). This was followed by the ReLU activation function which introduced non-linearity, enhancing the network’s ability to learn the complex pattern.

Following the initial convolutional process, the second convolutional operation was performed using a 1 × 1 kernel and regularized by L2 10^−3^. This convolutional primarily aids in increasing feature map depth without altering the spatial dimension of the data. Subsequently, a batch normalization layer was employed, which further assists in stabilizing the model by ensuring a normalized feature map before activation. Furthermore, shortcut path adjustments were configured; if the number of filters or stride did not match between the shortcut path and the output from the convolutional layers, the shortcut was adjusted with (1 × 1 convolution, stride = 1, same padding, and L2 10^−3^ followed by batch normalization) to match the main path’s stride and padding. This ensures the smooth addition of the shortcut, preserves essential information, and improves training stability. Finally, the output of the main path and adjusted shortcut were merged using an element-wise addition followed by a ReLU activation [38]. This combination allows for effective integration of features and enhances the stability and robustness of the training process. Moreover, max-pooling layers with a 2 × 2 pool size and stride of two were strategically positioned after certain convolutional blocks in the model to decrease the spatial dimension.

**Figure 4:**
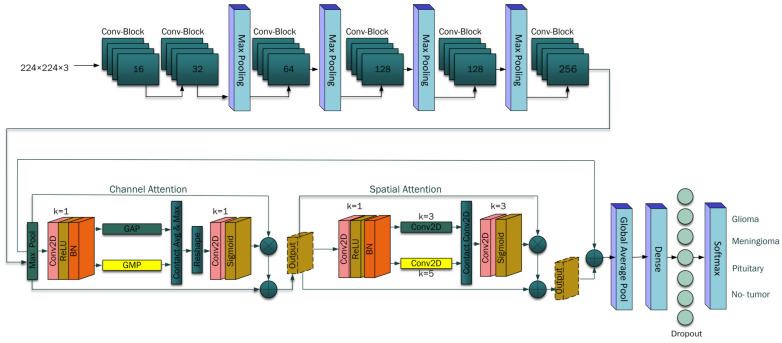
Proposed architecture for classification of brain tumors.

As shown in Figure 4, convolutional blocks involve increasing filter sizes: 16, 32, 64, 128, and 256 filters. After the max pooling layer, Algorithm 1 was employed to integrate the hybrid attention mechanism enhancing the model’s ability to recognize a relevant feature of brain tumor, the mechanism employed both channel and spatial attention, focusing the model processing capacity on the most informative parts of the features maps [39], which is essential for precise brain tumor classification. The architecture concluded with a robust classification head, which transforms the refined feature maps into a compact vector by utilizing a global average pooling layer. This vector feeds into a dense layer of 512 neurons, which is further processed with dropout for regularization [40,41].

In the end, a dense layer with softmax activation [41] was employed to determine the probability score for each class, classifying the decision labels as to whether the input images contained glioma, meningioma, pituitary, or no tumor cases. The pseudo-code for the hybrid attention mechanism is given below:
**Algorithm 1:** Pseudo-code for Hybrid Attention Mechanism*Input:*      •*F: Input feature map of dimension C × H × W*      •*Ratio: reduction factor in channel attention, set to 2**Output:*      •$F^{\prime \prime }$*: Refined output features map after applying hybrid attention**1.* Channel Attention*:**Reduce channel:* $F_{R}$ *= ReLU(BN(Conv(F, max(*$\frac{C}{ratio},1$*), 1 × 1)))**Pooling:* $A_{avg}$ *= GAP(*$F_{R}$*),*$\;A_{max}$ *= GMP(*$F_{R}$*)**Reshape:* $A_{F}$ *= Reshape(Concat(*$A_{avg},A_{max}$*), [1, 1, 2 × max(*$\frac{C}{ratio},1$*)])**Scale: S = Sigmoid(Conv(*$A_{F}$*, C, 1 × 1))**F′ = F*⊕*(F*⨂*S)**2.* *Spatial Attention:**Condense to single Channel: *$F_{C}\;$*= ReLU(BN(Conv(F′, 1, 1 × 1)))**Multi-Scale convolution: *$C_{1}=$*Conv(*$F_{C}$*, 1, 3 × 3,’ same’), *$C_{2}=$*Conv(*$F_{C}$*, 1, 5 × 5,’same’)**Attention map: A = Sigmoid(Conv(Concat(*$C_{1},C_{2}$*), 1, 3 × 3,’same’))**Apply: F″ = F′*⊕*(F′*⨂*A)**3.* *Combine with original input:**F″ = F*⊕*F″**Return F″*

### 3.3. Activation and Losses Functions

**Figure 5:**
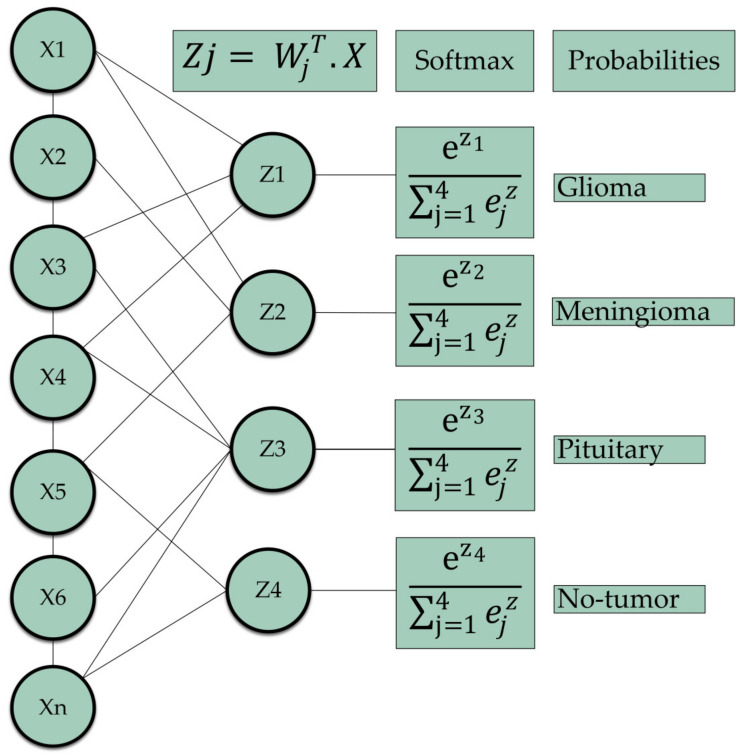
Depiction of the implementation of the softmax function as the output layer for the classification of brain tumors, where the input vector x is subjected to changes through hidden layers, which ultimately produce an output vector z that represents the score for each class. Subsequently, the softmax function transforms z into a probability distribution that encompasses the brain tumors.

The Rectified Linear Unit (ReLU) was employed in the presented framework as an activation function, which introduces non-linearity into a neural network architecture [38]. It processes an input value by returning the maximum between 0 and the input. This operation is mathematically desrcribed as follows:

(1) ReLU(x)=max(0,x)

where x is the input to the ReLU function. This setup ensures that positive inputs retain their original value, thereby maintaining their complete impact within the network. For inputs that are zero or negative, the function outputs zero, which effectively prevents negative values from affecting the subsequent layers of the network. The sigmoid function used in the attention mechanism normalizes scores to a range between 0 and 1. This normalization reflects the relative importance of the channel, enabling the network to prioritize the most significant feature of the task. The sigmoid function is denoted as follows:

(2) $$\sigma (x)=\frac{1}{1+e^{-x}}$$

where σ(x) has a characteristic sigmoid curve, e is the base of the natural logarithms, and x is the input variable. Moreover, the softmax function was applied at the output layer of the presented model. This function transforms a set of real values into a probability distribution over brain tumor classes. The mathematical expression for the softmax role is as follows:

(3) $$\sigma {\left(\vec{z}\right)}_{i}=\frac{e^{Z_{i}}}{\sum_{j=1}^{K}e^{Zj}}$$

where σ denotes softmax, $\vec{z}$ represents the input vector to function, $e^{Z_{i}}$ applies the standard exponential function to each element *i* of the input vector, and K denotes the total number of classes into which the inputs can be classified. Additionally, $e^{Zj}$ computes the exponential for each element *j* of the output vector, used in the denominator to normalize the results. Figure 5 illustrates the function of softmax as the output layer [41].

The categorical cross entropy was utilized for classification to measure the disparity between the predictions made by the algorithm’s actual values. The formulation of categorical cross entropy CE involves determining the error rate through the utilization of an equation.

(4) $$CE=-\sum_{i}^{N}y_{true}[i]\cdot \log(y_{pred}[i])$$

where $y_{true}[i]$ symbolizes the true class probabilities, $y_{pred}[i]$ represents the predicted probabilities of each class, and N is the number of classes.

### 3.4. Optimization Techniques

**Figure 6:**
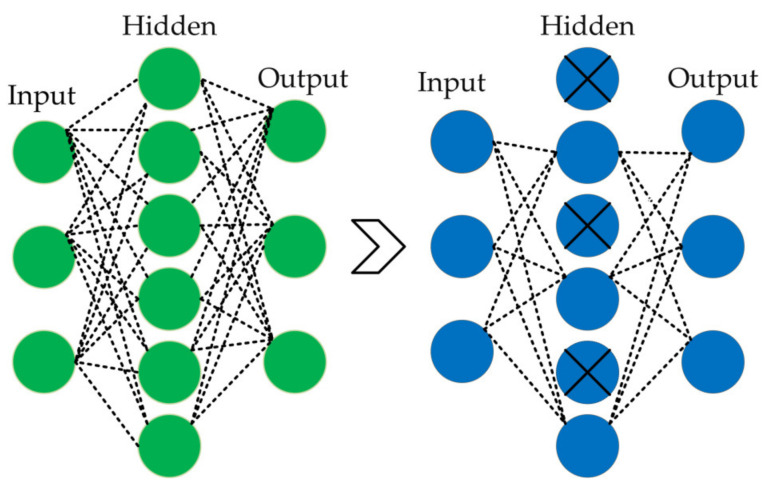
Visualization of a dropout layer on the right side, applying a 50% dropout rate.

The developed model employed various optimization techniques to specifically address the critical issue of overfitting in neural networks [42]. Overfitting is significant because it leads to a model that performs well on training data but does not generalize effectively to unseen data. To mitigate this, techniques such as dropout, L2 regularization, and ReduceLROnPlateau callbacks were incorporated. The dropout strategy is employed to selectively deactivate a portion of neurons wherein outputs were randomly set to zero during the training process [43]. This method reduces the model’s dependence on specific neurons, hence facilitating the development of a more robust feature representation and permitting a more general learning approach. By incorporating a 50% dropout rate, the model’s flexibility was enhanced and its ability to generalize effectively on unseen data was boosted. Figure 6 illustrates an example of a 50% dropout rate used in the proposed method.

L2 regularization [42], also known as weight decay, is employed in the neural network to mitigate the issue of overfitting and enhance performance. This technique was utilized in the proposed model due to its usefulness among the other regularization methods. The presented framework sets the hyperparameter 10^−3^ to regularization strength effectively. L2 regularization can be expressed as follows:

(5) $$\begin{array}{l} L2\;Regularization\;(weight\;decay)\\ Cost\;function=loss\;function+\lambda \sum_{i=1}^{N}\left|w_{i}^{2}\right| \end{array}$$

where λ is a hyperparameter that regulates the regularization strength, N represents the total number of parameters, $w_{i}$ signifies the *i*th parameters, and summation encompasses all parameters. The cost function combined with the loss represents the difference between the predictions and actual target values to form an objective function. The proposed model integrated the ReduceLROnPlateau callback with the Adam optimizer, as defined in Keras [44]. The callback is involved in dynamically adjusting the learning rate when a plateau in the target metric, such as validation loss. This adjustment ultimately enhanced the optimization process of the model. During the training process, it tracks the metric. If the metric does not demonstrate improvement over a predetermined number of epochs, the callback activates a reduction in the learning rate. The adjustment to the learning rate, denoted as $LR_{new}$, can be calculated using the following equation.

(6) $$LR_{new}=LR_{current}\times factor$$

where $LR_{current}$ denotes the learning rate of 0.001 before adjustment, and the factor represents the reduction factor that is applied to the learning rate set at 0.4 to prevent an excessive decrease in the learning rate and to ensure that the training process remains within operational limits.

### 3.5. Pre-Trained Models

Pre-trained neural networks, which have been trained on large-scale datasets like ImageNet that contain a wide range of image categories, have demonstrated their immense value in applications like as image classification and object recognition. These models are vastly proficient at analyzing intricate data patterns, facilitating their use as an initial framework for subsequent analytical tasks without requiring extensive training from scratch. The present study examined five pre-trained models, namely Xception [45], ResNet50V2, ResNet101V2 [46], DenseNet201, and DenseNet169 [47].

The Xception model improves the design of convolutional neural networks by substituting standard convolutions with depth-wise separable convolutions. This adjustment distinctly separates the process of spatial features and channel correlations into two phases. Initially, a pointwise convolution that modifies the dimension of the channel. Subsequently, depth-wise spatial convolution operates independently across each channel, thereby reducing the computational power and model complexity. The Residual Network (ResNet) architecture tackles the difficulties of training deep neural networks by including a residual learning framework. This method includes skip connections that help alleviate the problem of vanishing gradient. ResNet employed two primary types of blocks: identity blocks, which ensure dimensional consistency, and convolutional blocks, which adapt dimension as a requisite. ResNetV2 is an improved version that enhances the efficiency of identity mapping across skip connections, enhancing data transfer speed within blocks and offering variants like ResNet50V2 and ResNet101V2 with different layer counts to accommodate varying computational requirements.

Dense Convolutional Networks (DenseNets) utilize architectural features in which each layer is connected directly to subsequent layers in a feed-forward technique. DenseNets are structured into dense blocks. The pattern of these dense blocks varies between the models, such as DenseNet169 and DenseNet201, persuading their capacity for feature extraction. DenseNet169 comprises four dense blocks with layers distributed as 6, 12, 32, and 32, respectively. In contrast, DenseNet201 expands on the third block using a configuration of 6, 12, 48, and 32 layers. The arrangement of these blocks, coupled with downsampling, ensures that the model variant can optimally balance the depth and computational demand.

## 4. Experimental Results

The primary objective of this study is to perform classification on extensive data comprising 7023 MRI scans that illustrate glioma, meningioma, pituitary, and no-tumor cases. Classification development was achieved by incorporating the categorical cross-entropy loss function and softmax activation in order to achieve precise classification of MRI data. Initially, the data preparation involved resizing, labeling, and dividing data into 80% for training and 20% for testing with a random state value of 101, which was applied to shuffle the data effectively. The frameworks were trained over 50 epochs with eight batch sizes, including fivefold cross-validation [30] with Adam optimizer, and learning rate reduction was employed using the ReduceLROnPlateau callback to optimize the performance.

The platform employed well-known libraries, TensorFlow, Keras, Pandas, Numpy, Matplotlib, and Sklearn, facilitating the model building and analyzing data. For efficient training and optimization of models, the system included an NVIDIA GeForce GTX1080Ti GPU with Intel (R) Core (TM) i7-7800 CPU 3.5 GHz and 32 GB RAM. Python 3.7 was chosen as the programming language because of its comprehensive capabilities in data handling, analysis, and visualization. Algorithm 2 outlines the training and evaluation process.
**Algorithm 2:** 5-Fold Cross-Validation for Model Evaluation*1.* *Initialize Metrics collection**M*←*[] initialize list for evaluation metrics**2.* *5-Fold Cross-validation**D*←*Training data**For each* k ϵ{1,2,3,4,5}*:* *2.1.* *Data Division*${Train}_{k}$ *=*  $D-D_{k}$${Val}_{k}=D_{k}$ *2.2.* *Model Training**Train model using* ${Train}_{k}\;$*(D) and*${Val}_{k}(D_{k})$*Setup (callbacks and optimizer)* *2.3.* *Evaluate on testing set (T)*${temp}_{M}\leftarrow model.evaluate(T)$*Append* ${temp}_{M}$ *to M* *2.4.* *Compute Average Metrics**Final metrics*$\leftarrow \frac{1}{5}\sum_{k=1}^{5}M[k]$*3.* *Output Results**Final metrics hold the average values on the set T*

### 4.1. Evaluation Matrices

The effectiveness of the proposed framework was assessed using a range of measures. The framework employed precision, recall, F1-score, and accuracy for classification. These measures are crucial for evaluating the model’s ability to predict positive outcomes for various types of brain tumors accurately. Equations (7)–(10) provide the mathematical expressions for Precision, Recall, F1-score, and Accuracy.

(7) $$Precision=\frac{TP}{TP+FP}$$

(8) $$Recall=\frac{TP}{TP+FN}$$

(9) $$F1-Score=2\times \frac{Recall\times Precision}{Recall+Precision}$$

(10) $$Accuracy=\frac{TP+TN}{TP+TN+FP+FN}$$

Table 1 presents a comprehensive evaluation of both proposed and pre-existing models, highlighting a presented model improved with a hybrid attention mechanism. This advanced model achieved an exceptional accuracy of 98.33%, with precision and recall both at 98.30% and F1-score of 98.20%. In contrast, ResNet101V2 demonstrated suboptimal performance with an accuracy of 86.51%, precision of 86.10%, and recall and F1-score of 86.15%. The diminished efficacy of ResNet101V2 may be attributed to its distinct architectural attributes, which do not adequately accommodate the distinctive traits of the dataset employed in the study. The proposed model without attention also shows commendable results, attaining an accuracy of 96.97%, precision of 96.85%, recall of 96.75%, and F1-score of 96.80%, indicating robust base model capabilities. Furthermore, DenseNet169 outperformed other pre-trained architectures, achieving the highest metrics among them with an accuracy of 95.29%, precision and F1-score of 94.90%, and recall of 95.00%. Models such as DenseNet169, DenseNet201, and Xception showed better results compared to ResNet50V2. The models DenseNet169, DenseNet201, ResNet50V2, and ResNet101V2 were all trained using images with a size of 224 × 224 pixels. On the other hand, the Xception model was trained using images with a size of 299 × 299 pixels. In order to preserve the weights, the layers in these base models were kept non-trainable. The efficiency of the proposed model is evidenced by its training time of 460.17 s, indicating not only superior performance but also operational effectiveness compared to pre-trained models. The metrics clearly highlighted that the proposed model, particularly with the addition of the hybrid attention mechanism, is highly effective and demonstrates the potential for generalization across similar tasks.

### 4.2. Confusion Matrices

**Figure 7:**
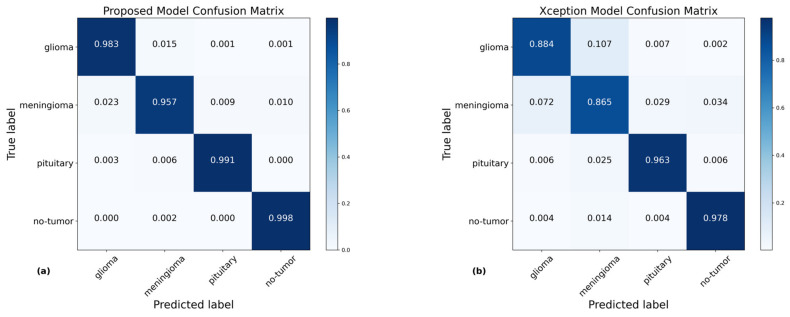

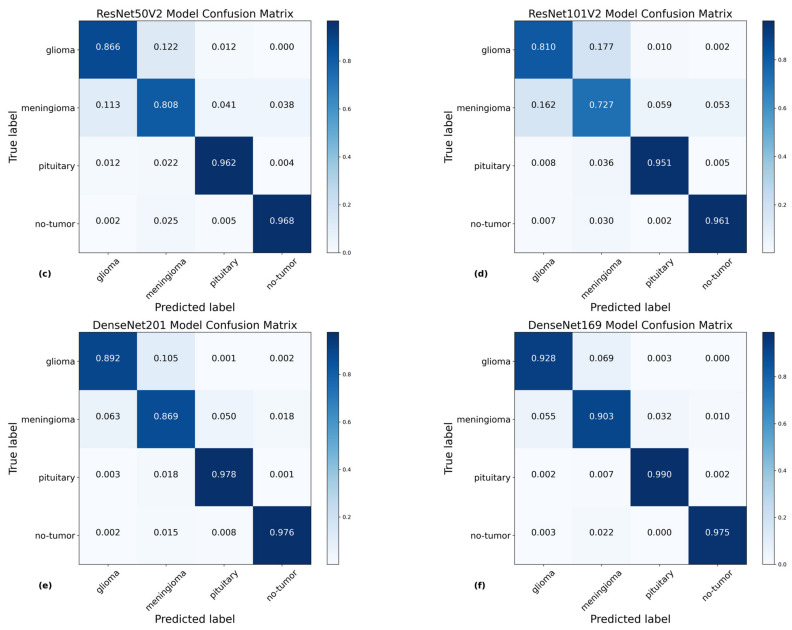
Illustration of the confusion matrix of the presented and pre-trained model using the testing data, showing the prediction score of each model. Specifically, (**a**) demonstrates that the proposed model with hybrid attention attained a high accuracy of 98.33%. In comparison, (**b**) indicates that the Xception model attained an accuracy of 92.64%, (**c**) shows the ResNet50V2 model achieved 90.39%, (**d**) reveals the ResNet101V2 model attained an accuracy of 86.51%, (**e**) displays the DenseNet201 model obtained an accuracy of 93.20%, and (**f**) highlights that the DenseNet169 achieved an accuracy of 95.29%.

A confusion matrix is an essential tool for evaluating classification methods [48]. The network developed in this study showed exceptional results in classifying different forms of brain tumors, consistently and correctly detecting each type during the testing phase. Figure 7 illustrates a visual comparison between the proposed mode and pre-trained models, highlighting the improved performance of the presented model. The findings demonstrate that the suggested method surpassed the performance of the pre-trained models with impressive accuracy scores: 98% for glioma, 96% for meningioma, 99% for pituitary tumors, and a flawless 100% for no-tumor cases. These accomplishments exceed the standards established by the presented model. Nevertheless, it is essential to recognize that the efficacy of glioma and meningioma falls behind the precision of the exceptional diagnostic prominence, demonstrating the pressing requirement for more study and thorough exploration in future investigations.

## 5. Discussion

This study introduces a novel methodology for evaluating the benchmark dataset, which consists of a comprehensive collection of 7023 primary brain tumor cases and normal brain cases. The proposed framework marked a significant advancement over methodologies that relied on extensive preprocessing and manual interventions to identify regions of interest. By reducing the need for complex preprocessing, the presented method not only simplifies the classification process but also enhances efficiency. Furthermore, Table 2 presents the results obtained from prior investigations that have examined similar brain tumor forms, although employing distinct methodologies for classification. Gumaei et al. proposed a hybrid approach that combined PCA, NGIST, and RELM. Although this hybrid method endeavored to capture an inclusive feature set, PCA might not consistently capture the non-linear pattern characteristic in MRI, possibly omitting essential tumor details and resulting in lower accuracy [18]. Swati et al. and Noreen et al. employed techniques that focused on improving generic architectures, particularly cutting-edge models [24,26]. The process of fine-tuning deep networks can take a significant amount of time. Due to the need to adjust numerous parameters in these enormous networks, the initiative process is arduous and requires a significant amount of resources. Perversely, the suggested approach is intentionally designed for brain tumor classification. The proposed approach effectively captures tumor-specific features while minimizing the processing requirements commonly concomitant with deep architectures.

Kaplan et al. primarily depend on traditional feature extraction methods, which are computationally challenging yet may inadvertently disregard subtle features and patterns in magnetic resonance (MR) images, resulting in lower accuracy [14]. Huang et al. developed the CNNBCN, a neural network architectural model that utilized a randomly generated graph approach, resulting in 95.49% classification accuracy [20]. Conversely, our methodology demonstrated enhanced classification capabilities. Ghassemi et al. investigated the domain of Generative Adversarial Networks (GANs) through the utilization of CNN-based GANs. Although GANs excel at generating synthetic pictures, their application in classification may include false subtleties that deviate from real-world MRI changes, thus compromising the accuracy of the classification [22].

Ayadi et al. developed a combination of DSURF-HOG and SVM for classification purposes. However, the method might not adequately address the hierarchical and spatial structures present in MRI images, areas where deep learning-based models demonstrate better performance [23]. Satyanarayana et al. utilized AMEA for noise reduction. They included these characteristics in a CNN with MCA in order to optimize the overall performance [27]. Similarly, Deepak et al. incorporated class weight focus loss into a Convolutional Neural Network (CNN) and employed the K-Nearest Neighbors (KNN) algorithm with the majority voting for optimal classification [28].

In contrast, the suggested approach demonstrates superior comparative performance. Furthermore, methods such as SURF-KAZE by Almalki et al. [49] and HOG-XG Boost by Shilaskar et al. [50] could face limitations in accurately capturing spatial and hierarchical patterns in MRI images, a domain where the deep learning model has shown strong capabilities, as evidenced by this study. Although the GAN-softmax method by Asiri et al. introduced several enhancements, it might demand more computational effort [51]. Contrarily, the suggested approach attained an impressive accuracy of 98.33% without relying on the preprocessing techniques. The model demonstrated strong performance directly on input images without the need for image manipulation, which makes it more adaptable and efficient in clinical settings.

**Table 2:** Comparative analysis of classification performance comparing the proposed method with the previous approach.

Authors	Dataset	Classes	Methods	Precision	Recall	F1-Score	Accuracy
Gumaei et al. [18]	Figshare 3064 Images	3	Hybrid PCA-NGIST-RELM	-	-	-	94.23
Swati et al. [26]	Figshare 3064 Images	3	VGG19-Fine tune	89.52	-	91.73	94.82
Kaplan et al. [14]	Figshare 3064 Images	3	NLBP-αLBP-KNN	-	-	-	95.56
Huang et al. [20]	Figshare 3064 Images	3	CNNBCN	-	-	-	95.49
Ghassemi et al. [22]	Figshare 3064 Images	3	CNN-based GAN	95.29	-	95.10	95.60
Ayadi et al. [23]	Figshare 3064 Images	3	DSURF-HOG-SVM	-	88.84	89.37	90.27
Noreen et al. [24]	Figshare 3064 Images	3	InceptionV3 Ensemble	93.00	92.00	92.00	94.34
Satyanarayana et al. [27]	Figshare 3064 Images	3	AMEA-CNN-MCA	-	-	-	94.00
Deepak et al. [28]	Figshare 3064 Images	3	CNN-MV-KNN	-	-	95.06	95.60
Almalki et al. [49]	Kaggle 2870 Images	4	SURF-KAZE-SVM	-	-	-	95.33
Asiri et al. [51]	Kaggle 2870 Images	4	GAN-Softmax	92.00	93.00	93.00	94.32
Shilaskar et al. [50]	Figshare, SARTAJ, Br35H 7023 Images	4	HOG-XG Boost	92.07	91.82.	91.85	92.02
Our work	Figshare, SARTAJ, Br35H, 7023 Images	4	CNN-Hybrid Attention	98.30	98.30	98.20	98.33

## 6. Conclusions

This study presented an advanced method for precise classification of several types of primary brain tumors, such as glioma, meningioma, pituitary, and no-tumor instances. The suggested techniques attained an outstanding accuracy of 98.33% by integrating a convolutional neural network with a hybrid attention mechanism. The proposed method improved the efficiency of brain tumor classification by reducing the feature extraction processes, resulting in a more streamlined diagnostic process. The results illustrate the suggested model’s exceptional ability to generalize, confirming its reliability and value in medical diagnostics. Moreover, it assists healthcare professionals in promptly and precisely identifying brain tumors. In the future, the aim is to enhance patient care by developing advanced systems that identify brain tumors in real-time and creating networks to analyze different forms of medical imaging in three dimensions.

## Figures and Tables

**Table 1:** Comparative analysis of the proposed and pre-trained models.

Models	Parameters	Precision	Recalls	F1-Score	Accuracy	Training Time(s)
Xception	22,963,756	92.35	92.20	92.25	92.64	1228.13
ResNet50V2	25,667,076	90.00	90.05	90.10	90.39	614.07
DenseNet201	20,293,188	92.95	92.75	92.85	93.20	1274.99
ResNet101V2	44,728,836	86.10	86.15	86.15	86.51	1035.39
DenseNet169	14,351,940	94.90	95.00	94.90	95.29	964.36
Proposed method without Attention	829,172	96.85	96.75	96.80	96.97	423.99
Proposed method with Attention	928,688	98.30	98.30	98.20	98.33	460.17

## Data Availability

The data will be available upon reasonable request from the corresponding author.
